# CXCR4-SDF-1 interaction potentially mediates trafficking of circulating tumor cells in primary breast cancer

**DOI:** 10.1186/s12885-016-2143-2

**Published:** 2016-02-19

**Authors:** M. Mego, D. Cholujova, G. Minarik, T. Sedlackova, P. Gronesova, M. Karaba, J. Benca, S. Cingelova, Z. Cierna, D. Manasova, D. Pindak, J. Sufliarsky, M. Cristofanilli, J. M. Reuben, J. Mardiak

**Affiliations:** 2nd Department of Oncology, Faculty of Medicine, Comenius University, Klenova 1, 833 10 Bratislava, Slovak Republic; Translational Research Unit, Faculty of Medicine, Comenius University, Bratislava, Slovakia; Institute of Molecular Biomedicine, Faculty of Medicine, Comenius University, Bratislava, Slovakia; Department of Pathology, Faculty of Medicine, Comenius University, Bratislava, Slovakia; National Cancer Institute, Bratislava, Slovakia; Cancer Research Institute, Slovak Academy of Sciences, Slovak Medical University, Bratislava, Slovakia; Slovak Medical University, Bratislava, Slovakia; Robert H Lurie Comprehensive Cancer Center, Northwestern University, Chicago, IL USA; Department of Hematopathology, The University of Texas MD Anderson Cancer Center Houston, Houston, TX USA

**Keywords:** Circulating tumor cells, CXCR4, SDF-1, Primary breast cancer, Cytokines, Chemokine receptors

## Abstract

**Background:**

Cytokines are involved in cancer invasion and metastasis. Circulating tumor cells (CTCs) play key role in tumor dissemination and are an independent survival predictor in breast cancer patients. The aim of this study was to assess correlation between CTCs and plasma cytokines in primary breast cancer (PBC) patients.

**Methods:**

This study included 147 chemotherapy naïve PBC patients. Peripheral blood mononuclear cells (PBMC) were depleted of hematopoetic cells using RossetteSep™ negative selection kit. RNA extracted from CD45-depleted PBMC was interrogated for expression of EMT (Twist1, Snail1, Slug, Zeb1) and epithelial (Ck19) gene transcripts by qRT-PCR. The concentrations of 51 plasma cytokines were measured using multiplex bead arrays.

**Results:**

CTCs were detected in 25.2 % patients. CTCs exhibiting only epithelial markers (CTC_EP) and only EMT markers (CTC_EMT) were present evenly in 11.6 % patients, while CTCs co-expressing both markers were detected in 2.0 % patients. Patients with presence of CTC_EP in peripheral blood had significantly elevated levels of plasma IFN-α2, IL-3, MCP-3, β-NGF, SCF, SCGF-β, TNF-β and SDF-1 compared to patients without CTC_EP. CTC_EP exhibited overexpression of SDF-1 receptor and CXCR4, but not other corresponding cytokine receptor, and in multivariate analysis SDF-1 was independently associated with CTC_EP. There was an inverse correlation between CTC_EMT and plasma cytokines CTACK, β-NGF and TRAIL, while presence of either subtype of CTCs was associated with increased level of TGF-β2.

**Conclusion:**

Using cytokine profiling, we identified cytokines associated with CTCs subpopulations in peripheral blood of PBC. Our data suggest that CXCR4-SDF-1 axis is involved in mobilization and trafficking of epithelial CTCs.

## Background

Metastatic cascade is a multistep process that enables the migration of tumor cells from the primary site to a distant location, where they can potentially establish a new cancer growth [1]. Circulating tumor cells (CTCs) are cancer cells that mediate tumor dissemination and play the key role in the metastatic cascade [1]. Numerous clinical trials showed the prognostic value of CTCs in primary as well as in metastatic breast cancer [2–5]. Increasing evidence suggest that CTCs are involved in tumor growths through the process termed self-seeding, defined as reinfiltration of the primary tumor or establishment of metastasis by more aggressive CTCs [6]. However, little is known about exact mechanism of migration and trafficking of CTCs in peripheral blood circulation.

Metastatic cascade is a highly inefficient process and only a very limited number of tumor cells that enter the blood circulation are capable of forming metastases [7–9]. Peripheral blood (PB) constitutes a highly unfavorable microenvironment for the CTCs owing to physical forces, the presence of immune cells, and anoikis, all of which collectively contribute to metastatic insufficiency [10–12]. At the same time, other components of peripheral blood including platelets, coagulation factors, various signaling molecules, growth factors and chemokines might protect CTCs and affect the selection of target organs for metastases formation [13].

Chemokines constitute a group of small molecular weight proteins that play an important role in physiological process like development, cell migration, and immunity as well as in many pathological conditions including cancer [14, 15]. The interactions between chemokines and their respective receptors help to regulate the trafficking and organization of cells within various tissue compartments [16]. An increasing body of evidence suggests that chemokines and their receptors play an important role in cancer progression and metastases formation. They are involved in several processes significant for cancer pathogenesis including epithelial to mesenchymal transition, tumor growth, angiogenesis and/or immunity [16, 17]. The expression of chemokines and their receptors are aberrant in different types of hematopoietic and solid tumors including breast cancer, and it is supposed that interaction between chemokines and their receptors could be involved in migration of cancer cells [16].

Composition of peripheral blood could reflect the activity and burden of the cancer disease. Presence of CTCs in PB is associated with poor prognosis in breast cancer, therefore we hypothesized that there are differences in the composition of peripheral blood based on the presence and CTCs. Previously we showed differences in the innate and adaptive immunity in inflammatory breast cancer patients according to presence of CTCs in PB [18]. We also showed that patients with CTCs in PB have an increased level of D-dimer, a marker of coagulation activation [19, 20]. In present study, we investigated the association between CTCs and peripheral blood cytokines with the intent of demonstrating which cytokines are involved in CTCs trafficking. To avoid the heterogeneity of metastatic sites having an effect on analyzed variables, we elected to study the primary breast cancer model.

## Methods

### Study patients

As a part of an ongoing translational study (Protocol TRU-SK 002; Study chair: M. Mego), 147 patients with stages I–III primary breast cancer (PBC) who were undergoing definitive surgery were included. From each patient we obtained peripheral blood to assess for the presence of CTCs as well as for the measuring cytokine/chemokine levels in plasma. The blood was drawn in the morning on the day of surgery and prior to surgical procedure. Each patient was given a complete diagnostic evaluation to exclude the presence of distant metastasis. Patients with concurrent malignancy other than non-melanoma skin cancer in the previous 5 years were excluded as well. In all patients, data regarding age, tumor stage, histology, regional lymph node involvement, hormone receptor status, and HER2 status were also recorded.

The study was approved by the Institutional Review Board (IRB) and Ethics Committee of the National Cancer Institute of Slovakia and was conducted between March 2012 and February 2013. Each participant provided written informed consent before study enrollment. Healthy donors (*N* = 60) were age-matched women without breast cancer who were recruited and consented according to the IRB-approved protocol.

### Detection of CTC in peripheral blood

CTC were detected in 5 mL of peripheral blood depleted of CD45 positive (CD45+) cells for CTC enrichment using a quantitative real-time polymerase chain reaction (qRT-PCR) assay, as described previously [18, 21].

#### RNA extraction and cell lines

Peripheral blood was subjected to CD45 depletion using the RossetteSep™ kit (StemCell Technologies, Vancouver, Canada), according to the manufacturer’s instructions. CD45-depleted cells were mixed with 300 μl of TRIzol® LS Reagent (Invitrogen Corporation, Carlsbad, CA) and stored at − 80 °C. The RNA was extracted from 250 μl of stored solution according to the manufacturer’s instructions. The precipitated pellet containing RNA was dissolved in 25 μl of nuclease-free water. All RNA preparation and handling steps took place in a laminar flow hood, under RNase-free conditions. RNA concentration was determined by absorbance readings at 260 nm (median = 5.95 ng/μl, range: 1.7–38.3 ng/μl). RNA extracted from HeLa, HCT 116, MCF7 and MDA-MB-231 cells were used as positive controls.

#### Identification of gene transcripts in CD45-depleted subsets

Isolated RNA was subjected to quantitative RT-PCR to detect EMT-inducing transcription factor (TF) gene transcripts (*TWIST1, SNAIL1, SLUG* and *ZEB1*) and epithelial antigen (KRT19). To detect cytokine receptors on CTCs, we analyzed expression of cytokine receptor genes in PB enriched for CTCs (CD45-depleted fraction). For each of tested cytokine receptor gene expression we analyzed 20 samples (10 CTC_EP positive and 10 CTCs negative). In brief, 1 μL of RNA were placed in 20 μL of reaction volume containing 10 μL of Maxima Probe/ROX qPCR Master Mix (Thermo Scientific), 0.15 μL QuantiFast RT Mix (Qiagen), 7.85 μL water and 1 μL of primers. The following TaqMan assays were purchased from LifeTechnologies (USA): *TWIST1*: Hs00361186_m1; *SNAIL1*: Hs00195591_m1; *SLUG*: Hs00161904_m1; *ZEB1*: Hs01566408_m1; *GAPDH*: Hs99999905_m1; *KRT19* Hs00761767_s1; *IFNAR2*: Hs01022060_m1; *IL3RA:* Hs00608141_m1*; CCR1*: Hs00174298_m1; *NTRK A*: Hs01021011_m1; c*-KIT:* Hs00174029_m1; *CXCR4:* Hs00607978_s1; *LTBR*: Hs00158922_m1. Amplicons or probes spanned intron–exon boundaries, with the exception of *KRT19* and *CXCR4*. Amplification was performed on an Roche LightCyler 480 Real-Time PCR system (Roche, Basel, Switzerland) using the cycling program: 50 °C for 30 min; 95 °C for 10 min; 40 cycles of 95 °C for 15 s and 60 °C for 60 s. All samples were analyzed in triplicate. Calibrator samples were run with every plate to ensure consistency of the PCR. For all fluorescence-based RT-PCR, fluorescence was detected between 0 and 40 cycles for the control and marker genes in single-plex reactions, which allowed for the deduction of the cycles at threshold (Ct) value for each product. Expression of the genes of interest was calibrated against expression of the housekeeping gene, *GAPDH*. Target cDNA was quantified using the delta-Ct method with the formula: 1 = 2 Ct(target-*GAPDH*).

#### CTC definition

Patient samples with higher *KRT19* gene transcripts than those of healthy donors were scored as epithelial CTCs positive (CTC_EP), while patient samples with higher EMT-TF (*TWIST1, SNAIL1, SLUG* and *ZEB1*) gene transcripts than those of healthy donors were scored as CTC_EMT positive. Expression of at least one of the markers (either epithelial or mesenchymal) at levels above the defined cutoff was sufficient to define a sample as CTC positive.

The highest expression levels of the *KRT19* and EMT-inducing TF gene transcripts relative to that of *GAPDH* were 3.4 × 10 ^−3^ (median 2.8 × 10^−6^, range: 0–3.4 × 10^−3^) for *KRT19*, 7.5 × 10^−4^ (median 0, range: 0–7.5 × 10^−4^) for *TWIST1*, 3.8 × 10^−2^ (median 3.1 × 10^−3^, range: 5.0 × 10^−4^–3.8 × 10^−2^) for *SNAIL1* and 1.7 × 10^−1^ (median 1.4 × 10^−2^, range: 2.2 × 10^−3^–1.7 × 10^−1^) for *ZEB1*, while *SLUG* transcripts were not detected in any of the samples from healthy donor. These highest expression values in healthy donors were used as “cutoff” to determine CTCs positivity.

### Plasma isolation

Venous PB samples were collected in EDTA-treated tubes in the morning on the day of surgery and centrifuged at 1000 g for 10 min at room temperature within 2 h of venipuncture and processed, as described previously [18]. Then, supernatants were collected and centrifuged at 1000 *g* for 10 min at room temperature to prevent cellular DNA contamination. Plasma samples were stored at −80 °C until further processing.

### Plasma cytokines and angiogenic factors analysis

Plasma samples were analyzed for 51 plasma cytokines and angiogenic factors: TGF-β1, TGF-β2, TGF-β3, IFN-α2, IL-1α, IL-2Rα, IL-3, IL-12p40, IL-16, IL-18, CTACK, Gro-α, HGF, LIF, MCP-3, M-CSF, MIF, MIG, β-NGF, SCF, SCGF-β, SDF-1α, TNF-β, TRAIL, IL-1β, Il-1RA, IL-2, IL-4, IL-5, IL-6, IL-7, IL-8, IL-9, IL-10, IL-12, IL-13, IL-15, IL-17, Eotaxin, FGF basic, G-CSF, GM-CSF, IFN-γ, IP-10, MCP-1, MIP-1α, MIP-1β, PDGF bb, RANTES, TNF-α, VEGF using pre-designed panels (Bio-Plex Pro TGF-β assay, Bio-Plex Pro Human Cytokine 21- and 27-plex immunoassays; Bio-Rad Laboratories, Hercules, CA, USA). Premixed cytokine standards and samples were diluted following manufacturer’s instructions and incubated with agitation (300 rpm, RT) with color-coded magnetic beads conjugated with monoclonal antibodies in the 96-well filter plate for 30 min (2 h for TGF-β assay). As all three TGF-β isoforms are secreted as inactive complexes, samples were first activated with 1 N HCl for 10 min, then neutralized with 1.2 N NaOH/0.5 M HEPES (Applichem, Darmstadt, Germany) and assayed immediately after neutralization step. Following 3 washes, samples were incubated with biotinylated detection antibody on a plate shaker (300 rpm agitation, RT) for 30 min in the dark (1 h for TGF-β). Each captured analyte was detected by the addition of streptavidin-phycoerythrin and quantified using a BioPlex suspension array reader (Bio-Rad Laboratories) equipped with 532 nm reporter laser and 635 nm classification laser diode. Cytokine concentrations (pg/ml) were calculated with Bio-Plex Manager 4.0 software using 5-parameter logistic (5PL) curve fitting.

### Statistical analysis

Patient characteristics were tabulated. The patients’ characteristics were summarized using the median (range) for continuous variables and frequency (percentage) for categorical variables. Normality of distribution was tested by the Kolmogorov-Smirnoff test. If normally distributed, sample means were tested by Student *t*-test or analysis of variance (ANOVA) with Bonferroni’s or Tamhane’s corrections, depending on homogeneity of variance in univariate analysis. Nonparametric Mann-Whitney *U* or Kruskal-Wallis H test were used for non-normally distributed data. Pearson’s or Spearman’s correlations were used according to the normality of data. Multivariate logistic regression analysis included CTC_EP (presence vs. absence), SDF-1 (continuous variable, the base-10 log-transformed due to significant non-normal distribution), hormone receptor status (positive for either or negative for both), HER-2 status (overexpressed or negative), tumor grade (1 and 2 vs. 3), tumor stage (T1 vs. ≥ T2) and N stage (N0 vs. N+), respectively. A backward model selection was conducted, and the final fitted model is shown in Table 5. All p values presented are two-sided, and associations were considered significant if the p value is less or equal to 0.05. Statistical analyses were performed using NCSS 2007 software (Hintze J, 2007, Kaysville, Utah, USA).

### Ethical approval

Institutional Review Board, National Cancer Institute, Bratislava, Slovakia, Protocol TRU-SK 002).

## Results

The study population consisted of 147 primary breast cancer patients with median age of 60 years (range: 35–83 years). Patients’ characteristics are shown in Table 1. There were 134 (91.2 %) patients with estrogen receptor positive (ER) and/or progesterone receptor positive (PR) tumors; 21 (14.3 %) patients with HER-2/neu amplified tumors. Majority of patients had tumor < 2 cm without axillary lymph nodes involvement, low/intermediate grade.

**Table 1:** Clinicopathology characteristics of patients

Variable	N	%
All	147	100.0
T-stage		
1	105	71.4
> 1	42	28.6
N-stage		
0	93	63.3
> 1	53	36.1
Grade		
1 and 2	89	60.5
3	56	38.1
Histology		
Invasive ductal carcinoma	127	86.4
Other	20	13.6
Hormone receptor status		
Negative for both	13	8.8
Positive for either	134	91.2
HER2/neu status		
Negative	126	85.7
Positive	21	14.3
Ki 67 (cut-off 14 %)		
Low	85	57.8
High	62	42.2
Molecular subtype		
Luminal A	85	57.8
Luminal B	30	20.4
HER2/neu positive	21	14.3
Triple negative	11	7.5
CTC_EP		
Present	20	13.6
Absent	127	86.4
CTC_EMT		
Present	20	13.6
Absent	127	86.4
Any CTC		
Present	37	25.2
Absent	110	74.8

### CTC detection

To determine overexpression of the EMT-inducing TF gene transcripts and *KRT19* in PBC patients, we compared the expression levels in patient samples with those of HDs. Totally, CTCs were detected in 37 (25.2 %) of patients. CTCs with only epithelial markers (CTC_EP) were present in peripheral blood of 17 (11.6 %) patients; CTC with EMT (CTC_EMT) only phenotype was present in 17 (11.6 %) of patients; in 3 (2.0 %) of patients, CTCs exhibited both epithelial and mesenchymal markers (Table 2). In one patient sample, there was overexpression of two EMT-inducing TF gene transcripts (*SLUG* and *TWIST1*), e.g. expression of both genes was higher than the cut-off value in the same sample.

**Table 2:** CTC detection and expression of the genes in CD45-depleted peripheral blood at levels higher than those of healthy donors

Gene	Number of positive samples	% of positive samples
*KRT19*	20	13.6
*TWIST1* ^a^	4	2.7
*SNAIL1*	0	0.0
*SLUG* ^a^	17	11.6
*ZEB1*	0	0.0
CTC_EP only	17	11.6
CTC_EMT only	17	11.6
CTC co-expressing both markers^b^	3	2.0
Any CTC	37	25.2

^a^In one patient sample, there was overexpression of two EMT-inducing TF gene transcripts (*SLUG* and *TWIST1*)

^b^In one sample there was co-overexpression of *KRT19* and *SLUG*, while in other two samples there was co-overexpression of *KRT19* and *TWIST1*

### Association between plasma cytokines and CTCs

Patients with presence of CTC_EP in peripheral blood had significantly elevated levels of plasma IFN-α2, IL-3, MCP-3, β-NGF, SCF, SCGF-β, SDF-1α, and TNF-β, as compared to patients without CTC_EP, while there was inverse correlation between CTC_EP and IL-1α (Table 3). Moreover there was a trend for positive association between CTC_EP and IL-2Ra (*p* = 0.06), MCP-1 (*p* = 0.08), IL-16 (*p* = 0.08) and TGF-β2 (*p* = 0.09).

**Table 3:** Association between CTCs and plasma cytokines

Cytokine	Mean	SEM	Median	*P*-value
IFN-α2 (ng/mL)				
CTC_EP -	100.52	2.01	98.45	0.002
CTC_EP+	116.29	4.84	112.74	
IL-3 (ng/mL)				
CTC_EP -	40.76	2.90	33.21	0.029
CTC_EP+	52.48	6.77	52.83	
MCP-3 (ng/mL)				
CTC_EP -	53.82	3.53	44.01	0.013
CTC_EP+	73.17	8.29	79.21	
β-NGF (ng/mL)				
CTC_EP -	1.25	0.18	0.86	0.002
CTC_EP+	1.52	0.44	1.31	
SCF (ng/mL)				
CTC_EP -	51.52	2.61	44.74	0.004
CTC_EP+	68.62	6.48	69.91	
SCGF-β (ng/mL)				
CTC_EP -	17377.49	820.65	15565.08	0.008
CTC_EP+	22633.38	2029.19	20271.15	
SDF-1α (ng/mL)				
CTC_EP -	223.75	9.35	219.53	0.036
CTC_EP+	265.86	22.85	262.79	
TNF-β (ng/mL)				
CTC_EP -	0.47	0.04	0.37	0.008
CTC_EP+	0.67	0.11	0.71	
CTACK (ng/mL)				
CTC_EMT -	1394.47	43.93	1343.50	0.046
CTC_EMT+	1185.51	110.71	1055.12	
β − NGF (ng/mL)				
CTC_EMT -	1.38	0.17	0.95	0.046
CTC_EMT+	0.77	0.43	0.77	
TRAIL (ng/mL)				
CTC_EMT -	50.57	2.83	44.37	0.044
CTC_EMT+	37.91	7.21	29.28	
TGF-β2 (ng/mL)				
Any CTC -	2099.93	61.43	2052.98	0.016
Any CTC+	2395.52	102.38	2227.53	

*Abbreviations*: *SEM* standard error of the mean, *CTC_EP –* epithelial circulating tumor cells absent, *CTC_EP+* epithelial circulating tumor cells absent, *CTC_EMT* mesenchymal circulating tumor cells absent, *CTC_EMT+,–* mesenchymal circulating tumor cells present

There were no positive association between CTC_EMT and plasma cytokines; however, patients with CTC_EMT had significantly decreased plasma levels of CTACK, β − NGF and TRAIL, as compared to patients without CTC_EMT as well as a trend for inverse association between CTC_EMT and SDF-1 (*p* = 0.06), IFN-α2 (*p* = 0.06) and IL-3 (*p* = 0.08) (Table 3). Moreover, patients with any CTCs (epithelial or mesenchymal) in peripheral blood had significantly elevated level of TGF-β2 and decreased level of IL-1α (Table 3).

### Expression of cytokine receptors in peripheral blood enriched for CTCs

**Fig. 1:**
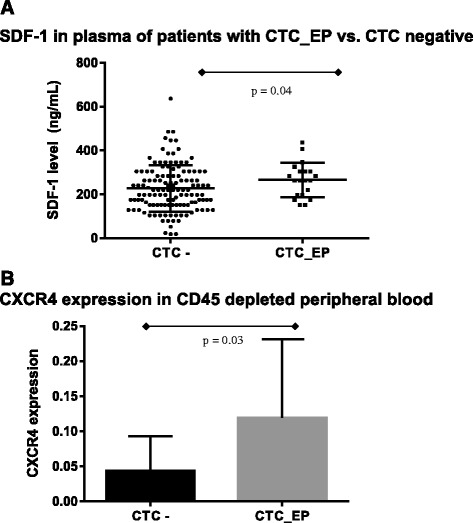
**a** SDF-1 in plasma of patients with none CTCs vs. CTC_EP (mean ± SEM = 223.75 ± 9.35 vs. 265.86 ± 22.85, *p* = 0.04). **b** Expression of CXCR4 (SDF-1 receptor) in peripheral blood enriched for CTCs (CD45 depleted) in patients with none CTCs vs. CTC_EP (relative expression ± SEM = 0.0433 ± 0.0275 vs. 0.1191 ± 0.0814, *p* = 0.03)

Based on an observed association between CTC_EP and cytokines, the following corresponding cytokine receptors were detected in PB enriched for CTC_EP (CD45 depleted PB with *KRT19* overexpression); IFNAR2 (interferon-alpha/beta receptor beta chain) for IFN-α2, IL3RA (interleukin 3 receptor alpha) for IL-3, CCR1 (chemokine (C-C motif) receptor 1) for MCP-3, NTRK A (tropomyosin receptor kinase A) for β-NGF, c-KIT (mast/stem cell growth factor receptor) for SCF and SCGF-β, CXCR4 for SDF-1α and LTBR (lymphotoxin beta receptor) for TNF-β. CTC_EP samples had a significantly higher expression of CXCR4 compared with those of CTC negative samples (Table 4, Fig. 1), while there was no statistically significant difference in any of the remaining corresponding cytokines receptors (Table 4).

**Table 4:** Expression of chemokine receptors on CTC_EP

Chemokine receptor	CTC_EP –	CTC_EP+	*p*-value
CCR1			
Mean	0.0052	0.0077	0.23
SEM	0.0018	0.0018	
Median	0.0038	0.0055	
CXCR4			
Mean	0.0433	0.1191	0.03
SEM	0.0275	0.0275	
Median	0.0275	0.0814	
IFNAR2			
Mean	0.0083	0.0149	0.15
SEM	0.0033	0.0033	
Median	0.0075	0.0110	
LTBR			
Mean	0.0027	0.0033	0.36
SEM	0.0008	0.0008	
Median	0.0018	0.0030	
c-KIT			
Mean	0.0007	0.0006	0.85
SEM	0.0002	0.0002	
Median	0.0005	0.0006	
NTRK1			
Mean	0.0005	0.0012	0.23
SEM	0.0003	0.0003	
Median	0.0004	0.0008	
IL3RA			
Mean	0.0530	0.0735	0.60
SEM	0.0162	0.0162	
Median	0.0511	0.0559	

*Abbreviations*: *SEM* standard error of the mean, *CTC_EP –* epithelial circulating tumor cells absent, *CTC_EP+* epithelial circulating tumor cells absent

### Multivariate analysis of association between SDF-1 and Epithelial CTCs

In univariate analysis, we found association of several plasma cytokines and CTC_EP; however, only chemokine receptor for SDF-1 cytokine (CXCR4) was detected in PB enriched for CTC_EP. Therefore, we build multivariate regression model to analyze if SDF-1 is associated with CTC_EP independently of known prognostic factors including T and N stage, hormone and HER-2/neu receptor status and tumor grade, respectively (Table 5). This analysis confirmed that plasma SDF-1 level is independently correlated with CTC_EP in peripheral blood.

**Table 5:** Multivariate logistic regression model for the binary indicator of CTC_EP

Variable	Odds ratio	95 % CI Low	95 % CI Upper	*P*-value
SDF-1	16.90	0.91	314.67	0.03
(continous variable, log)				
T-stage	1.03	0.32	3.38	0.97
T1 vs. ≥ T2				
N-stage	0.88	0.30	2.60	0.81
N0 vs. N+				
Grade	0.62	0.21	1.82	0.38
1 and 2 vs. 3				
ER/PR status	3.13	0.76	12.96	0.13
Negative for both vs. positive for either				
HER2/neu status	1.64	0.33	8.20	0.52
Negative vs. overexpressed				

## Discussion

In this prospective translational study, we showed for the first time an association between different subsets of CTCs and plasma cytokines in primary breast cancer patients. Several plasma cytokines positively correlated with presence of epithelial CTCs in the peripheral blood, while CTC_EMT phenotype inversely correlated with CTACK, β-NGF and TRAIL. Interestingly, an increased plasma level of TGF-β2 positively correlated with presence of any subset of CTCs. To determine which cytokines are involved in CTCs trafficking, we evaluated expression of cytokine receptors in PB enriched for CTC_EP, corresponding to cytokines that are positively associated with CTC_EP. Among seven evaluated receptors, only CXCR4, receptor for SDF-1, was overexpressed in this PB fraction implicating that the CXCR4-SDF-1 axis is mainly involved in trafficking and homing of epithelial CTCs. Multivariate analysis showed that SDF-1 is independently associated with CTC_EP, as previously reported [22], stressing the significance of CXCR4-SDF-1 axis for epithelial CTCs [23]. Numerically, other chemokine receptors seemed to be overexpressed on CTC_EP; therefore, we can’t exclude the involvement of these receptors in the trafficking and homing of CTCs, as well. Due to chemokine promiscuity other chemokine receptors might also elaborate in these processes [24].

Previously, numerous studies suggest importance of CXCR4-SDF-1 axis in tumor dissemination and cancer progression [25–27]. CXCR4 overexpression in tumor tissue was associated with inferior outcome in several cancers including breast cancer [28]. Moreover, expression of CXCR4 on cancer cells was associated with cancer stem cell phenotype and treatment resistance [29]. Previously, it was showed that CXCR4 is involved in homing of breast cancer cells predominantly to bone and liver [30, 31], however, this is a first study that revealed CXCR4 overexpression in the PB enriched for epithelial CTCs, and first study that showed clear association between plasma level of CXCR4 ligand SDF-1 and subset of CTCs with epithelial phenotype. In previous study, we correlated SDF-1 expression in tumor tissue with presence of CTCs (manuscript submitted), and there was no correlation between epithelial CTCs and expression of SDF-1 in primary tumor tissue, nor correlation between plasma and tissue SDF-1 level (data not shown), suggesting, that source of plasma SDF-1, that attracts CTCs into circulation, is outside the primary breast tumor. CXCR4 antagonists, were first evaluated in the treatment of HIV, later was discovered their potential for mobilizing CD34+ hematopoietic peripheral stem cells, but these classes of drugs are promising anticancer drugs and several clinical trials are ongoing including breast cancer [23, 32–34]. Recently it was shown, that CXCR4 signaling regulates breast cancer stem cell activities and thus could be important in tumour formation at the sites of metastases. *In vitro*, it was observed a greater reduction in self-renewal following CXCR4 inhibition in the CXCR4 over-expressing cells compared to cells without CXCR4 overexpression [35]. Based on observed data, we suggest, that CXCR4 antagonist could affect mobilization and trafficking of CTCs as well as self-renewal capacity of cancer cells and thus could represent promising drugs in breast cancer. At the same time, we suppose that expression of CXCR4 on CTCs could represent a novel marker for selection of anti-CXCR4 therapy [22].

Beyond the role in CTCs trafficking, elevated plasma levels of IFN-α2, IL-3, MCP-3, β-NGF, SCF, SCGF-β, TNF-β and SDF-1α in patients with CTC_EP could reflect more aggressive disease and/or activation of immune system as a host reaction to CTCs in peripheral blood. In particular, IFN-α2, IL-3, and TNF-β are cytokines involved in inflammation and anticancer immunity, and their positive association with CTC_EP might be due to activation of anticancer immunity. However, many tumors including breast cancer may endorse immune tolerance to facilitate tumor growth utilizing different cytokines including SCF and SCGF-β that are responsible for recruiting and/or activating immune suppressor cells, such as myeloid-derived suppressor cells [36]. Other cytokines including β-NGF might promote tumor growth through regulating breast cancer stem cell self-renewal and plasticity [37].

We observed inverse correlation between CTC_EMT and plasma cytokines CTACK, β-NGF and TRAIL. EMT is a transdifferentiation process that plays an important role in tumor invasion and release of CTCs into the peripheral circulation. Beside its role in CTCs generation, EMT is closely related to immunity and induces impaired dendritic cells and T-regulatory cells, suggesting an immunosuppressive effect of EMT, as showed previously [17, 38]. Therefore, an inverse association between observed cytokines and CTC_EMT phenotype might reflect EMT induced immunosuppression. Tumor growth factor β2 (TGF-β2) correlated with presence of any subset of CTCs (CTC_EP and/or CTC_EMT) in peripheral blood. Although TGF-β2 works as a tumor suppressor during cancer initiation, it may act as a tumor promoter during tumor progression and is involved in multiple processes in tumor progression such as in inducing of EMT [39]. Previously, we showed abnormalities in the innate and adaptive immunity of IBC patients with ≥1 CTCs and ≥5 CTCs per 7.5 mL of peripheral blood and observed that an association between cytokines and CTCs might suggest impaired activity of the immune system in patients with CTCs in peripheral blood [12].

Numerous methods are currently utilized for CTCs detection and characterization capable to determine different subpopulations of CTCs with different clinical and biological value [11]. Therefore, all data regarding CTCs should be interpreted within the context of the detection method used. In this study, we detected CTCs indirectly, by qRT-PCR method, based on expression of *KRT19* and EMT-TFs respectively. We defined CTC_EP based on expression of *KRT19*, an epithelial marker, commonly utilized for CTCs detection with established prognostic value in breast cancer [5]. However, due to CTCs heterogeneity, it remains to determine the associations between SDF-1 cytokine and other subsets of epithelial CTCs that lack *KRT19* overexpression. In our study, we used four EMT-TFs for detection of CTC_EMT, known to play a role in breast cancer pathogenesis [17, 20, 40–43]. We observed overexpression of two of them (SLUG and TWIST1) in some patients, while detection of SNAIL1 and ZEB1 doesn’t contributed to CTCs detection. We hypothesize, that this could be due to background expression of SNAIL1 and ZEB1 in CD45 depleted peripheral blood, that mask possible signal from CTCs overexpressing these two EMT related genes.

## Conclusion

In conclusion, the results of this prospective translational study suggest that specific cytokines, particularly the CXCR4-SDF-1 axis might be involved in the trafficking and homing of CTCs or alternatively, increased levels of these cytokines could be a marker of more aggressive disease. Thus, therapeutic inhibition of this signaling pathway could represent a novel therapeutic target interfering with tumor dissemination and metastatic cascade in primary breast cancer patients.

## Abbreviations

ANOVA: analysis of variance
CCR1: chemokine (C-C motif) receptor 1
CD45+: CD45 positive
c-KIT: mast/stem cell growth factor receptor
CTC_EMT: CTC with EMT phenotype
CTC_EP: CTC with epithelial
CTCs: circulating tumor cells
ECM: extracellular matrix
EMT: epithelial-to-mesenchymal transition
EMT-TF: EMT-inducing transcription factors
ER: estrogen receptor
HD: healthy donors
IFNAR2: interferon-alpha/beta receptor beta chain
IL3RA: interleukin 3 receptor alpha
IRB: Institutional Review Board
LTBR: lymphotoxin beta receptor
MBC: metastatic breast cancer
NTRK A: tropomyosin receptor kinase A
PB: peripheral blood
PBC: primary breast cancer
PBMC: peripheral blood mononuclear cells
PR: progesterone receptor
qRT-PCR: quantitative real time polymerase chain reaction
SEM: standard error of the mean
